# Relationship between rs7586085, *GALNT3* and *CCDC170* gene polymorphisms and the risk of osteoporosis among the Chinese Han population

**DOI:** 10.1038/s41598-022-09755-z

**Published:** 2022-04-12

**Authors:** Jiaqiang Zhang, Qinlei Cai, Wangxue Chen, Maoxue Huang, Renyang Guan, Tianbo Jin

**Affiliations:** 1 Department of Medical Image, People’s Hospital of Wanning, Wanning, Hainan China; 2 Department of Radiology, Hainan Hospital Affiliated to Hainan Medical College, Haikou, Hainan China; 3 Key Laboratory of Resource Biology and Biotechnology in Western China, Ministry of Education, Northwest University, Xi’an, Shaanxi 710069 China; 4 Provincial Key Laboratory of Biotechnology of Shaanxi Province, Northwest University, Xi’an, China

**Keywords:** Diseases, Health care

## Abstract

Osteoporosis (OP) has plagued many women for years, and bone density loss is an indicator of OP. The purpose of this study was to evaluate the relationship between the polymorphism of the rs7586085, *CCDC170* and *GALNT3* gene polymorphisms and the risk of OP in the Chinese Han population. Using the Agena MassArray method, we identified six candidate SNPs on chromosomes 2 and 6 in 515 patients with OP and 511 healthy controls. Genetic model analysis was performed to evaluate the significant association between variation and OP risk, and meanwhile, the multiple tests were corrected by false discovery rate (FDR). Haploview 4.2 was used for haplotype analysis. In stratified analysis of BMI ˃ 24, rs7586085, rs6726821, rs6710518, rs1346004, and rs1038304 were associated with the risk of OP based on the results of genetic models among females even after the correction of FDR (*q*^*d*^ < 0.05). In people at age ≤ 60 years, rs1038304 was associated with an increased risk of OP under genetic models after the correction of FDR (*q*^*d*^ < 0.05). Our study reported that *GALNT3* and *CCDC170* gene polymorphisms and rs7586085 are the effective risk factors for OP in the Chinese Han population.

## Introduction

Osteoporosis (OP) is one of the most common and impactful metabolic diseases of elders^1^ with the clinical features of the reduced bone mineral density (BMD) and bone structure destruction leading to an increased risk of fracture^2^. Age and sex are the two most relevant hazard factors for OP^3^. Elders, especially in postmenopausal women, are at a high risk for it due to accelerated bone loss^2,3^. It has been 8.9 million fractures worldwide for the increasing and prevalence of OP^4^. Patients with brittle fractures are hospitalized more than 400,000 times a year and treated 2.5 million times a year, which are placed a huge financial burden on patients and their families^5^. It is genetic and environmental factors that contribute to OP^6,7^. Twin studies have shown a BMD heritability of 0.51 to 0.76 for different bones. Previous genome-wide association analyses (GWASs) have identified more than 60 loci related to bone density and OP, many of which are thought to play important roles in bone, such as RANKL, OPG, ESR1, and LRP5^2^. GWASs have identified certain SNPs at risk for OP^8^.

*GALNT3,* located in 2q24-31, encodes UDP-*N*-acetyl-ɑ-d-galactosamine-polypeptide: polypeptide *N*-acetylgalactosaminyltransferase-3 (ppGalNacT3)^9^, and initiates the glycosylation of O-GalNAC. A GWAS by Duncan et al. covered an association between the *GALNT3* gene and BMD and fracture risk in postmenopausal women^10^. The substances encoded by the *GALNT3* gene in the human body are mainly involved in bone metabolism and related processes^11^. Studies have shown that *GALNT3* gene mutation can cause hyperphosphatemic familial tumoral calcinosis that is an autosomal recessive genetic disease and that can lead to the symptoms of hyperphosphatemia^12,13^. Moreover, the abnormalities of ppGalNacT3 can cause a disorder of phosphorus regulation, thereby affecting bone mineralization and BMD, which is one of the most important indicative indexes of primary osteoporotic fractures^14^. GWASs have been confirmed that it has been very successful in identifying common genetic variations related to bone density. A GWAS study of BMD found that *CCDC170* was strongly associated with BMD^15^. *CCDC170* encodes the protein *CCDC170*, which is a predicted protein containing the coiled helical domain (CCDC), is associated with the golgi body, stabilizes peri-nuclear microtubules (MTs), and plays an vital role in the known process of mt-dependent golgi structure^16^.

In this study, we selected samples from Chinese Han ethnicity from Xi’an 630 Hospital and People's Hospital of Wanning to study the relationship between rs7586085, *GALNT3* (rs6726821, rs6710518, and rs1346004) and *CCDC170* (rs4869739 and rs1038304) gene polymorphisms and the OP phenotype in postmenopausal women in China. These findings are expected to elucidate important new pathways in bone metabolism and to contribute to the development of new therapies, which may have prognostic value.

## Materials and methods

### Study participants

The case–control study was collected from hospitals included 515 patients with OP and 511 healthy controls from April 2019 to April 2020. Subjects with OP were recruited from the Xi’an 630 Hospital, Yanliang, Xi’an, Shaanxi, China and People's Hospital of Wanning, Hainan Province, China. The control group was those who went to the two hospitals for general inspection, who had no history of cancer or any disease related to bone organs. BMD at the lumbar spine (l2-4) and femoral neck of all subjects were determined using a dual-energy X-ray absorptiometry (lunar specialist 1313). We diagnosed OP in strict accordance with the criteria of the World Health Organization^17^.

### Clinical data and demographic information

We used standardized epidemiological questionnaires, including area of residence, age, sex, BMI, ethnicity, and family history, to collect personal data in face-to-face interviews. The 5 mL venous blood was taken from each subject for DNA extraction. All the volunteers signed an informed consent that stated the purpose of this study and the experiment. The protocol (approved number: hnwnrmyy-2020-yxk-05) was approved by the ethics committee of People's Hospital of Wanning, and was in accordance with the Declaration of Helsinki.

### SNPs selection and genotyping

We selected carefully rs7586085, *GALNT3* and *CCDC170* SNPs from 1000 Genomes Project (http://www.internationalgenome.org/) and the SNPs were in conformity with the minor allele frequency (MAF) ˃ 5%. The distribution of SNPs genotypes in the control group was in accordance with Hardy–Weinberg equilibrium (HWE) (*p* > 0.05). We genotyped SNPs using Agena MassARRAY RS1000. Moreover, the call rate of our results was greater than 95%. Ten percent of the samples were genotyped repeatedly and the concordance rate was 100%. The investigators who genotyped the samples were unknown the status of the sample. Then, using the Haploview 4.2, the pairwise linkage disequilibrium (LD) of rs7586085, *GALNT3* and *CCDC170* gene polymorphisms was estimated. After finished the steps mentioned above, we selected six SNPs rs7586085, rs6726821, rs6710518, rs1346004, rs4869739 and rs1038304 as the gene variation to study. Genomic DNA was extracted from peripheral blood with the Gold Mag-Mini genomic DNA purification kit (Gold Mag Co. Ltd., Xi’an, China) and quantified with the Nano Drop spectrophotometer 2000C (Thermo Scientific, Waltham, Massachusetts, USA). SNPs genotyping of the Agena MassARRAY RS1000 instrument (Shanghai, China) system was performed in accordance with the standard scheme recommended by the manufacturer. The experimental data were managed and analyzed using Agena Typer 4.0 software. Primers of each SNP are presented in Supplementary Table S1.

### Statistical analyses

First, the HWE of each SNP in the control group was inspected by the goodness-of-fit chi-square test. In this study, all *p* values were bilateral, and *p* value less than 0.05 was regarded as the cut-off value, which was considered statistically significant. The chi-square test was used to compare the allele frequency and genotype frequency of each SNP in the patients and controls. Odds ratio (OR) and 95% confidence interval (95% CI) were obtained by unconditional logistic regression analysis adjusted for BMI and age. To account for multiple comparisons at each genetic model, we further considered FDR adjusted *p* value (*q*^*d*^) < 0.05 as significance. The relationship between genotypes and OP risk was tested in different genetic models (co-dominant, dominant, recessive, and additive) using PLINK 1.9. The demographic characteristics were experimented using SPSS statistical software package, version 19.0 (SPSS Inc., Chicago, Illinois, USA). Haploview 4.2 was used to perform the LD and haplotype analysis of these six polymorphisms to OP risk.

### Ethical approval

All procedures completed in this study were in keeping with the ethical standards of the ethics committee of People's Hospital of Wanning and with the 1964 Helsinki declaration and its later amendments.

## Results

### Population characteristics

A total of 515 female patients with OP and 511 female controls were enrolled in our study. The mean age [± standard deviation (SD)] of the case group was 63.72 ± 5.58 years at diagnosis and that of the control group was 62.87 ± 4.68 years at recruitment.

### SNPs and OP risk

The essential information and allele frequencies of *GALNT3* and *CCDC170* gene polymorphisms and rs7586085 are displayed in Table 1. The six SNPs were all conformed to the HWE without deviation in the control group. The minor allele of each SNP was considered as a risk factor. Results of the four genetic models analyses are shown in Table 2. We used logistic regression to analyze SNPs of four genetic models. The results showed that there were no significant loci.

**Table 1:** Basic information of six SNPs in this study.

SNP ID	Gene	Chr	Position	Alleles A/B	MAF				Role	HWE *p-*value	OR (95% CI)	*p*^a^
					n	Case	n	Control				
rs7586085	–	2	166577489	G/A	401	0.390	379	0.372	–	0.395	1.08 (0.90–1.29)	0.408
rs6726821	*GALNT3*	2	166578114	G/T	401	0.389	379	0.372	Intronic	0.394	1.08 (0.90–1.29)	0.408
rs6710518	*GALNT3*	2	166583244	T/C	365	0.359	363	0.359	Intronic	0.847	1.00 (0.83–1.20)	0.994
rs1346004	*GALNT3*	2	166601046	A/G	401	0.389	383	0.375	Intronic	0.258	1.06 (0.89–1.27)	0.519
rs4869739	*CCDC170*	6	151901802	A/T	238	0.231	211	0.206	Intronic	0.893	1.16 (0.94–1.42)	0.178
rs1038304	*CCDC170*	6	151933175	G/A	475	0.461	442	0.433	Intronic	0.177	1.12 (0.94–1.33)	0.205

*SNP* single nucleotide polymorphism, *Chr* chromosome, *Alleles A/B* Minor/Major alleles, *HWE* Hardy–Weinberg equilibrium, *MAF* minor allele frequency, *OR* odds ratio, *95% CI* 95% confidence interval, *n* the number of minor allele.

*p* < 0.05 indicates statistical significance.

^a^Pearson Chi-squared test.

**Table 2:** Genotypic model analysis of the relationship between SNPs and the risk of osteoporosis.

SNP ID	Model	Genotype	Case	Control	With adjusted	
					OR (95% CI)	*p*
rs7586085	Co-dominant	A/A	187 (36.4%)	205 (40.3%)	1.00	
		G/A	253 (49.2%)	229 (45.0%)	1.22 (0.93–1.60)	0.145
		G/G	74 (14.4%)	75 (14.7%)	1.10 (0.75–1.61)	0.627
	Dominant	A/A	187 (36.4%)	205 (40.3%)	1.00	
		G/A-G/G	327 (63.6%)	304 (59.7%)	1.19 (0.92–1.53)	0.177
	Recessive	A/A-G/A	440 (85.6%)	434 (85.3%)	1.00	
		G/G	74 (14.4%)	75 (14.7%)	0.98 (0.69–1.40)	0.929
	Additive	–	–	–	1.09 (0.91–1.30)	0.364
rs6726821	Co-dominant	T/T	188 (36.5%)	206 (40.4%)	1.00	
		G/T	253 (49.1%)	229 (44.9%)	1.22 (0.93–1.59)	0.146
		G/G	74 (14.4%)	75 (14.7%)	1.09 (0.75–1.60)	0.629
	Dominant	T/T	188 (36.5%)	206 (40.4%)	1.00	
		G/T-G/G	327 (63.5%)	304 (59.6%)	1.19 (0.92–1.53)	0.179
	Recessive	T/T-G/T	441 (85.6%)	435 (85.3%)	1.00	
		G/G	74 (14.4%)	75 (14.7%)	0.98 (0.69–1.39)	0.928
	Additive	–	–	–	1.09 (0.91–1.30)	0.366
rs6710518	Co-dominant	C/C	189 (37.2%)	206 (40.8%)	1.00	
		T/C	273 (53.7%)	235 (46.5%)	1.27 (0.98–1.66)	0.075
		T/T	46 (9.1%)	64 (12.7%)	0.81 (0.52–1.24)	0.323
	Dominant	C/C	189 (37.2%)	206 (40.8%)	1.00	
		T/C-T/T	319 (62.8%)	299 (59.2%)	1.17 (0.91–1.51)	0.217
	Recessive	C/C-T/C	462 (90.9%)	441 (87.3%)	1.00	
		T/T	46 (9.1%)	64 (12.7%)	0.70 (0.47–1.05)	0.087
	Additive	–	–	–	1.01 (0.84–1.22)	0.917
rs1346004	Co-dominant	G/G	188 (36.5%)	205 (40.2%)	1.00	
		A/G	253 (49.1%)	227 (44.5%)	1.22 (0.94–1.60)	0.139
		A/A	74 (14.4%)	78 (15.3%)	1.05 (0.72–1.53)	0.809
	Dominant	G/G	188 (36.5%)	205 (40.2%)	1.00	
		A/G-A/A	327 (63.5%)	305 (59.8%)	1.18 (0.92–1.52)	0.203
	Recessive	G/G-A/G	441 (85.6%)	432 (84.7%)	1.00	
		A/A	74 (14.4%)	78 (15.3%)	0.94 (0.66–1.33)	0.714
	Additive	–	–	–	1.07 (0.89–1.28)	0.478
rs4869739	Co-dominant	T/T	309 (60.0%)	321 (62.8%)	1.00	
		A/T	174 (33.8%)	169 (33.1%)	1.07 (0.82–1.39)	0.604
		A/A	32 (6.2%)	21 (4.1%)	1.50 (0.84–2.67)	0.167
	Dominant	T/T	309 (60.0%)	321 (62.8%)	1.00	
		A/T-A/A	206 (40.0%)	190 (37.2%)	1.12 (0.87–1.44)	0.376
	Recessive	T/T-A/T	483 (93.8%)	490 (95.9%)	1.00	
		A/A	32 (6.2%)	21 (4.1%)	1.46 (0.83–2.58)	0.188
	Additive	–	–	–	1.14 (0.92–1.40)	0.222
rs1038304	Co-dominant	A/A	144 (28.0%)	156 (30.6%)	1.00	
		G/A	267 (51.8%)	266 (52.2%)	1.11 (0.83–1.47)	0.489
		G/G	104 (20.2%)	88 (17.3%)	1.33 (0.92–1.91)	0.130
	Dominant	A/A	144 (28.0%)	156 (30.6%)	1.00	
		G/A-G/G	371 (72.0%)	354 (69.4%)	1.16 (0.88–1.52)	0.284
	Recessive	A/A-G/A	411 (79.8%)	422 (82.7%)	1.00	
		G/G	104 (20.2%)	88 (17.3%)	1.24 (0.91–1.71)	0.178
	Additive	–	–	–	1.15 (0.96–1.37)	0.138

*p* < 0.05 indicates statistical significance.

OR (95% CI) and *p* values were calculated by logistic regression analysis with adjustments for BMI and age.

*SNP* single nucleotide polymorphism, *OR* odds ratio, *95% CI* 95% confidence interval.

We collected the height and weight of the individuals. Then, stratified analysis was performed whether BMI was greater than 24 (Table 3). Individuals with BMI > 24 were considered overweight. Stratified analysis by BMI indicated that the rs7586085 polymorphism was significantly related to an increased risk of OP in BMI ˃ 24 (heterozygote: OR 2.13, 95% CI 1.28–3.57, *p* = 0.004, *q*^*d*^ = 0.036; additive: OR 1.55, 95% CI 1.10–2.18, *p* = 0.012, *q*^*d*^ = 0.048; alleles: OR 1.49, 95% CI 1.07–2.07, *p* = 0.018, *q*^*d*^ = 0.05). The polymorphism of rs6726821 was significantly associated with an increased risk of OP in BMI ˃ 24 (heterozygote: OR 2.13, 95% CI 1.28–3.57, *p* = 0.004, *q*^*d*^ = 0.029; alleles: OR 1.55, 95% CI 1.07–2.07, *p* = 0.018, *q*^*d*^ = 0.046). The polymorphism of rs6710518 was significantly associated with an increased risk of OP in BMI ˃ 24 (heterozygote: OR 2.11, 95% CI 1.27–3.51, *p* = 0.004, *q*^*d*^ = 0.024; dominant: OR 2.02, 95% CI 1.25–3.28, *p* = 0.004, *q*^*d*^ = 0.021). The polymorphism of rs1346004 was significantly associated with an increased risk of OP in BMI ˃ 24 (heterozygote: OR 2.13, 95% CI 1.28–3.57, *p* = 0.004, *q*^*d*^ = 0.018; dominant: OR 2.10, 95% CI 1.29–3.41, *p* = 0.003, *q*^*d*^ = 0.036; additive: OR 1.55, 95% CI 1.10–2.18, *p* = 0.012, *q*^*d*^ = 0.043; alleles: OR 1.49, 95% CI 1.07–2.07, *p* = 0.018, *q*^*d*^ = 0.043). The polymorphism of rs1038304 was significantly associated with an increased risk of OP in BMI ˃ 24 (homozygote: OR 2.41, 95% CI 1.18–4.91, *p* = 0.016, *q*^*d*^ = 0.048; recessive: OR 2.24, 95% CI 1.19–4.19, *p* = 0.012, *q*^*d*^ = 0.039). After FDR correction, significant association remained among rs7586085, rs6726821, rs6710518, rs1346004, rs1038304 and increased risk of OP. Rs4869739 polymorphism was not observed significance with OP in BMI ˃ 24 after FDR correction.

**Table 3:** Association between SNPs and OP after stratification by BMI under different genotypic models.

SNP	Model	Genotype	BMI ˃ 24					BMI ≤ 24				
			case	control	OR (95% CI)	*p*	*q*^*d*^	case	control	OR (95% CI)	*p*	*q*^*d*^
rs7586085	Co-dominant	A/A	51	58	1			136	70	1		
		G/A	95	52	2.13 (1.28–3.57)	**0.004**	**0.036**	158	84	0.97 (0.66–1.44)	0.880	0.960
		G/G	31	19	2.01 (1.00–4.04)	**0.049**	0.093	43	27	0.82 (0.47–1.44)	0.487	0.923
	Dominant	A/A	51	58	1			136	70	1		
		G/A-G/G	126	71	2.10 (1.30–3.41)	**0.003**	0.108	201	111	0.93 (0.64–1.35)	0.716	0.859
	Recessive	A/A-G/A	146	110	1			294	154	1		
		G/G	31	19	1.31 (0.70–2.50)	0.402	0.467	43	27	0.83 (0.50–1.40)	0.491	0.884
	Additive	–	–	–	1.55 (1.10–2.18)	**0.012**	**0.048**	–	–	0.92 (0.71–1.20)	0.543	0.815
	Alleles	G/A	157	90	1.49 (1.07–2.07)	**0.018**	0.050	244	138	0.92 (0.71–1.20)	0.541	0.847
rs6726821	Co-dominant	T/T	51	58	1			137	70	1		
		G/T	95	52	2.13 (1.28–3.57)	**0.004**	**0.029**	158	84	0.96 (0.65–1.42)	0.849	0.955
		G/G	31	19	2.01 (1.00–4.04)	**0.049**	0.088	43	27	0.81 (0.46–1.43)	0.471	0.997
	Dominant	T/T	51	58	1			137	70	1		
		G/T-G/G	126	71	2.10 (1.29–3.41)	**0.003**	0.054	201	111	0.93 (0.64–1.34)	0.686	0.852
	Recessive	T/T-G/T	146	110	1			295	154	1		
		G/G	31	19	1.31 (0.69–2.47)	0.402	0.452	43	27	0.83 (0.49–1.40)	0.483	0.966
	Additive	–	–	–	1.55 (1.10–2.18)	0.051	0.083	–	–	0.92 (0.70–1.19)	0.520	0.851
	Alleles	G/T	157	90	1.49 (1.07–2.07)	**0.018**	**0.046**	244	138	0.92 (0.70–1.19)	0.519	0.890
rs6710518	Co-dominant	C/C	52	58	1			137	70	1		
		T/C	100	54	2.11 (1.27–3.51)	**0.004**	**0.024**	173	86	1.03 (0.69–1.51)	0.888	0.940
		T/T	24	17	1.72 (0.82–3.61)	0.149	0.215	22	24	0.47 (0.25–0.90)	**0.023**	0.414
	Dominant	C/C	52	58	1			137	70	1		
		T/C-T/T	124	71	2.02 (1.25–3.28)	**0.004**	**0.021**	195	110	0.91 (0.63–1.31)	0.606	0.873
	Recessive	C/C-T/C	152	112	1			310	156	1		
		T/T	24	17	1.12 (0.57–2.21)	0.737	0.737	22	24	0.46 (0.25–0.85)	**0.014**	0.504
	Additive	–	–	–	1.49 (1.05–2.13)	**0.026**	0.055	–	–	0.80 (0.59–1.06)	0.123	0.554
	Alleles	T/C	148	88	1.40 (1.00–1.96)	**0.047**	0.094	217	134	0.82 (0.63–1.07)	0.144	0.576
rs1346004	Co-dominant	G/G	51	58	1			137	70	1		
		A/G	95	52	2.13 (1.28–3.57)	**0.004**	**0.018**	158	83	0.97 (0.66–1.44)	0.897	0.923
		A/A	31	19	2.01 (1.00–4.04)	**0.049**	0.084	43	29	0.76 (0.43–1.31)	0.321	0.825
	Dominant	G/G	51	58	1			137	70	1		
		A/G-A/A	126	71	2.10 (1.29–3.41)	**0.003**	**0.036**	201	112	0.92 (0.63–1.33)	0.649	0.899
	Recessive	G/G-A/G	146	110	1			295	153	1		
		A/A	31	19	1.31 (0.69–2.47)	0.402	0.439	43	29	0.77 (0.46–1.28)	0.307	0.850
	Additive	–	–	–	1.55 (1.10–2.18)	**0.012**	**0.043**	–	–	0.89 (0.69–1.16)	0.400	0.960
	Alleles	A/G	157	90	1.49 (1.07–2.07)	**0.018**	**0.043**	244	141	0.89 (0.69–1.16)	0.400	0.900
rs4869739	Co-dominant	T/T	98	81	1			211	114	1		
		A/T	61	41	1.24 (0.75–2.04)	0.406	0.430	113	64	0.96 (0.65–1.40)	0.819	0.951
		A/A	18	7	1.78 (0.69–4.55)	0.232	0.298	14	4	1.86 (0.59–5.79)	0.285	0.855
	Dominant	T/T	98	81	1			211	114	1		
		A/T-A/A	79	48	1.32 (0.82–2.11)	0.250	0.310	127	68	1.01 (0.70–1.47)	0.959	0.959
	Recessive	T/T-A/T	159	122	1			324	178	1		
		A/A	18	7	1.65 (0.65–4.15)	0.290	0.348	14	4	1.89 (0.61–5.83)	0.269	0.880
	Additive	–	–	–	1.29 (0.89–1.87)	0.182	0.252	–	–	1.07 (0.77–1.48)	0.681	0.908
	Alleles	A/T	97	55	1.39 (0.95–2.03)	0.085	0.128	141	72	1.07 (0.78–1.47)	0.681	0.876
rs1038304	Co-dominant	A/A	48	42	1			96	67	1		
		G/A	87	69	1.12 (0.66–1.91)	0.666	0.685	180	89	1.43 (0.95–2.14)	0.083	0.427
		G/G	42	18	2.41 (1.18–4.91)	**0.016**	**0.048**	62	26	1.68 (0.96–2.93)	0.066	0.396
	Dominant	A/A	48	42	1			96	67	1		
		G/A-G/G	129	87	1.37 (0.82–2.26)	0.227	0.303	242	115	1.49 (1.01–2.18)	**0.043**	0.387
	Recessive	A/A-A/G	135	111	1			276	156	1		
		G/G	42	18	2.24 (1.19–4.19)	**0.012**	**0.039**	62	26	1.35 (0.82–2.23)	0.236	0.850
	Additive	–	–	–	1.48 (1.05–2.08)	**0.025**	0.056	–	–	1.32 (1.01–1.73)	**0.042**	0.504
	Alleles	G/A	171	105	1.36 (0.98–1.88)	0.062	0.097	304	141	1.29 (0.99–1.68)	0.053	0.382

Bold type *p* < 0.05 indicates statistical significance.

*q*^*d*^: FDR-adjusted p value.

The FDR adjustment was conducted at each taxonomic level.

*SNP* single nucleotide polymorphism, *OR* odds ratio, *95% CI* 95% confidence interval.

We also investigated the relationship of six SNPs with OP risk under age subgroup. As summarized in Table 4, the polymorphism of rs1038304 was found to significantly increase the risk of OP at age ≤ 60 years even after FDR correction (homozygote: OR 2.99, 95% CI 1.50–6.00, *p* = 0.002, *q*^*d*^ = 0.024; dominant: OR 2.49, 95% CI 1.43–4.34, *p* = 0.001, *q*^*d*^ = 0.036; additive: OR 1.73, 95% CI 1.23–2.44, *p* = 0.002, *q*^*d*^ = 0.018; alleles: OR 1.64, 95% CI 1.21–2.23, *p* = 0.001, *q*^*d*^ = 0.018). There was no significant association observed in other SNPs.

**Table 4:** Association between SNPs and OP after stratification by age under different genotypic models.

SNP	Model	Genotype	Age ˃ 60 years					Age ≤ 60 years				
			case	control	OR (95% CI)	*p*	*q*^*d*^	case	control	OR (95% CI)	*p*	*q*^*d*^
rs7586085	Co-dominant	A/A	131	136	1			56	69	1		
		G/A	187	130	1.51 (1.09–2.10)	**0.015**	0.135	66	99	0.89 (0.54–1.46)	0.642	1.101
		G/G	49	51	1.02 (0.64–1.63)	0.918	0.972	25	24	1.35 (0.67–2.71)	0.399	1.026
	Dominant	A/A	131	136	1			56	69	1		
		G/A-G/G	236	181	1.37 (1.01–1.87)	**0.045**	0.270	91	123	0.98 (0.61–1.57)	0.936	0.936
	Recessive	A/A-G/A	318	266	1			122	168	1		
		G/G	49	51	0.82 (0.54–1.26)	0.364	0.819	25	24	1.44 (0.76–2.73)	0.260	1.337
	Additive	–	–	–	1.11 (0.89–1.39)	0.341	0.818	–	–	1.09 (0.78–1.53)	0.599	1.078
	Alleles	G/A	285	232	1.10 (0.88–1.37)	0.395	0.711	116	147	1.05 (0.77–1.44)	0.756	0.972
rs6726821	Co-dominant	T/T	131	137	1			57	69	1		
		G/T	187	130	1.52 (1.09–2.11)	**0.013**	0.234	66	99	0.87 (0.53–1.42)	0.566	1.132
		G/G	49	51	1.03 (0.65–1.64)	0.894	0.975	25	24	1.31 (0.66–2.63)	0.441	0.992
	Dominant	T/T	131	137	1			57	69	1		
		G/T-G/G	236	181	1.38 (1.02–1.89)	**0.040**	0.288	91	123	0.95 (0.60–1.52)	0.846	0.923
	Recessive	T/T-G/T	318	267	1			123	168	1		
		G/G	49	51	0.82 (0.54–1.26)	0.373	0.746	25	24	1.42 (0.75–2.69)	0.276	1.104
	Additive	–	–	–	1.12 (0.90–1.39)	0.320	0.823	–	–	1.08 (0.77–1.50)	0.670	1.049
	Alleles	G/T	285	232	1.11 (0.89–1.38)	0.371	0.788	116	147	1.04 (0.76–1.42)	0.810	0.941
rs6710518	Co-dominant	C/C	132	137	1			57	69	1		
		T/C	201	135	1.55 (1.12–2.15)	**0.008**	0.288	72	100	0.94 (0.57–1.52)	0.784	0.941
		T/T	29	41	0.76 (0.45–1.30)	0.318	0.881	17	23	0.89 (0.42–1.89)	0.756	0.972
	Dominant	C/C	132	137	1			57	69	1		
		T/C-T/T	230	176	1.37 (1.00–1.87)	**0.047**	0.212	89	123	0.92 (0.58–1.48)	0.743	0.991
	Recessive	C/C-T/C	333	272	1			129	169	1		
		T/T	29	41	0.60 (0.36–0.99)	**0.046**	0.237	17	23	0.92 (0.46–1.87)	0.821	0.924
	Additive	–	–	–	1.07 (0.84–1.36)	0.577	0.799	–	–	0.94 (0.66–1.33)	0.724	1.002
	Alleles	T/C	259	217	1.05 (0.84–1.31)	0.670	0.828	106	146	0.93 (0.68–1.27)	0.647	1.059
rs1346004	Co-dominant	G/G	131	136	1			57	69	1		
		A/G	187	129	1.52 (1.09–2.12)	**0.013**	0.156	66	98	0.87 (0.53–1.43)	0.587	1.112
		A/A	49	53	0.98 (0.62–1.55)	0.936	0.936	25	25	1.27 (0.64–2.54)	0.491	1.040
	Dominant	G/G	131	136	1			57	69	1		
		A/G-A/A	236	182	1.36 (1.00–1.86)	**0.049**	0.196	91	123	0.95 (0.60–1.52)	0.846	0.923
	Recessive	G/G-A/G	318	265	1			123	167	1		
		A/A	49	53	0.78 (0.51–1.20)	0.259	0.777	25	25	1.37 (0.73–2.59)	0.322	1.054
	Additive	–	–	–	1.09 (0.88–1.36)	0.424	0.727	–	–	1.07 (0.77–1.48)	0.707	1.018
	Alleles	A/G	285	235	1.08 (0.87–1.35)	0.475	0.724	116	148	1.03 (0.75–1.40)	0.864	0.889
rs4869739	Co-dominant	T/T	218	190	1			91	131	1		
		A/T	120	115	0.92 (0.67–1.27)	0.622	0.829	54	54	1.32 (0.81–2.17)	0.264	1.188
		A/A	29	14	1.75 (0.90–3.43)	0.101	0.331	3	7	0.54 (0.13–2.19)	0.387	1.161
	Dominant	T/T	218	190	1			91	131	1		
		A/T-A/A	149	129	1.01 (0.75–1.38)	0.931	0.958	57	61	1.22 (0.76–1.97)	0.411	0.986
	Recessive	T/T-A/T	338	305	1			145	185	1		
		A/A	29	14	1.81 (0.93–3.49)	0.079	0.284	3	7	0.49 (0.12–1.98)	0.319	1.148
	Additive	–	–	–	1.10 (0.86–1.41)	0.441	0.722	–	–	1.09 (0.72–1.64)	0.700	1.050
	Alleles	A/T	178	143	1.11 (0.86–1.43)	0.423	0.693	60	68	1.18 (0.80–1.74)	0.397	1.099
rs1038304	Co-dominant	A/A	119	94	1			25	62	1		
		G/A	184	170	0.86 (0.61–1.21)	0.391	0.741	83	96	2.31 (1.29–4.13)	0.005	**0.036**
		G/G	64	54	0.94 (0.60–1.49)	0.798	0.927	40	34	2.99 (1.50–6.00)	**0.002**	**0.024**
	Dominant	A/A	119	94	1			25	62	1		
		G/A-G/G	248	224	0.88 (0.63–1.22)	0.445	0.668	123	130	2.49 (1.43–4.34)	**0.001**	**0.036**
	Recessive	A/A-A/G	303	261	1			108	158	1		
		G/G	64	54	1.04 (0.69–1.55)	0.864	0.972	40	34	1.67 (0.96–2.90)	0.068	0.408
	Additive	–	–	–	0.95 (0.76–1.19)	0.672	0.834	–	–	1.73 (1.23–2.44)	**0.002**	**0.018**
	Alleles	G/A	312	278	0.95 (0.77–1.18)	0.654	0.851	163	164	1.64 (1.21–2.23)	**0.001**	**0.018**

Bold type *p* < 0.05 indicates statistical significance.

*q*^*d*^: FDR-adjusted p value.

The FDR adjustment was conducted at each taxonomic level.

*SNP* single nucleotide polymorphism, *OR* odds ratio, *95% CI* 95% confidence interval.

### Association of haplotype with OP

**Figure 1:**
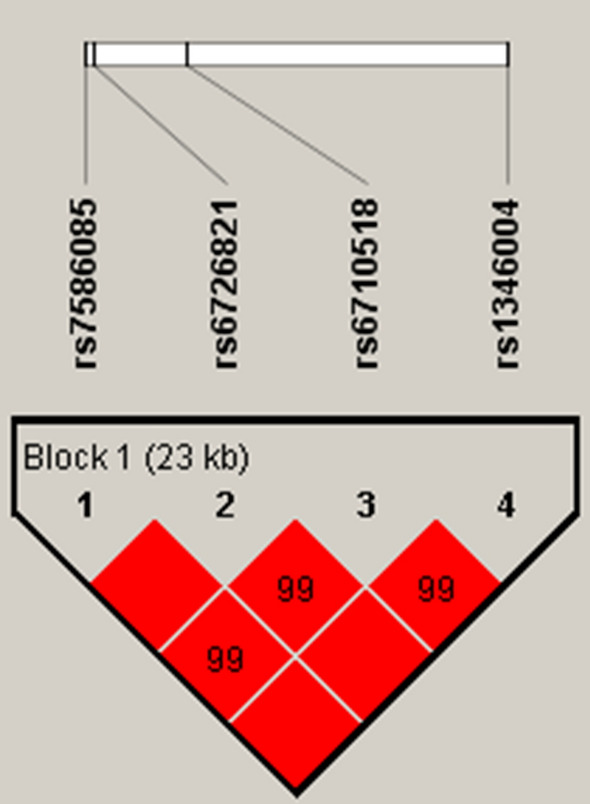
Linkage disequilibrium (LD) analysis of four SNPs. The block structure was assessed using Haploview 4.2.

We further explored the LD and haplotype analyses of those SNPs. A haplotype block with strong LD is presented in Fig. 1 with four SNPs including rs7586085, rs6726821, rs6710518, and rs1346004. The distribution of frequencies for haplotypes in the cases and controls are observed in Table 5. The haplotype results show a remarkable associations of ‘GGCA’ haplotypes with an increased risk of OP (OR 2.74, 95% CI 1.20–6.22, *p* = 0.016) (Table 5).

**Table 5:** Haplotype frequencies of polymorphisms and their association with the risk of OP.

Haplotype	Freq (case)	Freq (control)	*p*^*a*^	Crude		With adjusted	
				OR (95% CI)	*p*	OR (95% CI)	*p*
**Block: rs7586085|rs6726821|rs6710518|rs1346004**							
GGTA	0.368	0.364	0.842	1.02 (0.85–1.23)	0.837	1.03 (0.85–1.24)	0.760
GGCA	0.021	0.008	**0.011**	2.80 (1.24–6.35)	**0.014**	2.74 (1.20–6.22)	**0.016**
ATCG	0.389	0.377	0.550	1.06 (0.88–1.26)	0.550	1.06 (0.89–1.27)	0.496

Bold type *p* < 0.05 indicates statistical significance.

*OR* odds ratio, *95% CI* 95% confidence interval.

^a^Two-sided χ^2^ test/Fisher's exact tests.

## Discussion

The main characteristic of OP is that it can decrease the risk in bone density. BMD is defined as the amount of minerals in bone and is associated with estrogen^18^. The majority of BMD-related SNPs identified by GWASs are located in non-coding regions of the genome^2^. Our study provides an extensive evidence that SNPs (rs7586085, rs6726821, rs6710518, rs1346004, rs4869739, and rs1038304) located on chromosomes 2 and 6 can serve as multiple loci which were associated with an increased risk of OP. We demonstrated that risk SNPs loci were significantly associated with an increased OP risk in various genetic models, and haplotype ‘GGCA’ consisting of four SNPs was also associated with increasing the risk of OP. Additionally, it turns out that all the five SNPs, which were associated with increasing the risk of OP in people with BMI ˃ 24, were obviously some risk loci in overweight people. Rs1038304 was associated with an increased risk of OP in people at age ≤ 60 years. The six SNPs what we studied were in the non-coding region of the gene. Rs7586085 was close to the *GALNT3* gene, but located on an unknown gene.

In a meta-analysis, gender- and age-adjusted variants of the *CCDC170/ESR1* gene were found to be associated with BMD^19^. Other studies found that rs1038304 polymorphism on *CCDC170* gene was associated with fracture and vertebral fracture risk in postmenopausal women in China^20^. *CCDC170* gene polymorphism may not only play an important role in bone metabolism. Previous studies have found a significant association between vertebral fracture risk and rs1038304 and a protective effect^20^, and other study has found that rs1038304 is related to BMD^21^. Therefore, we studied the relationship between *CCDC170* gene polymorphism and OP risk, and found that the SNPs were associated with increasing the risk of OP. Rs1038304 was in the intron region of *CCDC170* gene and was associated with increasing the risk of OP.

Previous studies have suggested a relationship between *GALNT3* gene polymorphism and the OP phenotype in postmenopausal women in China^9^. *GALNT3* is an enzyme involved in the glycosylation of serine and threonine residues, whose process is critical to the integrity and viability of fibroblast growth factor-23 (*FGF23*). A functional copy of *GALNT3* may be sufficient to secrete complete *FGF23* and appropriately regulate serum phosphate^22^. *FGF23* is a phosphorus-promoting hormone produced by bones which enhances the reabsorption of calcium and sodium into the kidney^23^. The polymorphisms of *GALNT3* and *FGF23* can cause familial neoplastic calcinosis in hyperphosphatemia^24^. Furthermore, Runx2 is an important transcription factor for chondrocyte maturation^25^. *GALNT3* is one of the downstream genes of Runx2 in chondrocytes, however many GALNT family genes are expressed in cartilage tissue. *Galnt3* mice showed short stature and shortened limbs. *GALNT3* has non-redundant function during chondrocyte maturation^25^. Ichikawa et al. found increased bone density *Galnt3*-deficient mice^22^. Generally speaking, polymorphism in the *GALNT3* gene plays an important role in BMD loss. A significant relationship between the polymorphism of rs6710518 and BMD has been discovered^9^. Therefore, polymorphisms of *GALNT3* gene were detected to be the risk factor to OP, leading to new findings on the pathological mechanism of OP.

Although we successfully identified individual trait correlations and pleiotropic SNPs of OP, our study still had some potential limitations. First, we only found some polymorphisms in some of the non-coding genes on chromosome 2 and chromosome 6, and it may have other polymorphisms around. Second, the sample size of the case and control groups was small, which was only limited to the population of Northwest China. Therefore, we need to continue to expand the sample size and further study the mechanisms at the cellular level and in vivo.

## Conclusion

Taken together, our study uncovered a new association between genetic polymorphisms on chromosomes 2 and 6 and the risk of OP in the Chinese Han population. These outcomes are helpful to further study the mechanism of polymorphism affecting the pathogenesis of OP. The larger sample sizes were, the more cellular and in vivo studies to further explore and confirm the function of these polymorphisms in increasing the risk of femoral OP were needed, which will provide new insights on prevention and treatment of OP.

## Supplementary Information

Supplementary Table S1.

## Data Availability

The data that support the findings of this study are available on request from the corresponding author.
