# Supplementary material for: Relationship between rs7586085, GALNT3 and CCDC170 gene polymorphisms and the risk of osteoporosis among the Chinese Han population
Source: Sci Rep. 2022 Apr 12;12:6089. doi: 10.1038/s41598-022-09755-z (PMC9005502; doi:10.1038/s41598-022-09755-z)
Supplement: Supplementary file 1 — Supplementary Table S1. [file 41598_2022_9755_MOESM1_ESM.docx]

**Supplementary Table S1** Primers used for this study

| SNP_ID | 2nd - PCRP | 1st - PCRP | UEP-DIR | UEP SEQ |
| --- | --- | --- | --- | --- |
| rs7586085 | ACGTTGGATGCAAGG  CAACAAGCATTGAAC | ACGTTGGATGATTCC  CGTGGTTCCCTTATC | R | CATAAAAGGA  TTTGCATTGTG |
| rs6726821 | ACGTTGGATGTGTGTACT  GTCATGCTCGTG | ACGTTGGATGAAG  AATAGCAAGACTTGGAC | R | ccgaAAGACTT  GGACTAACCC |
| rs6710518 | ACGTTGGATGACA  CTAGAGCCTTTGGACAG | ACGTTGGATGACA  CTAGAGCCTTTGGACAG | F | CCTTTGGAC  AGTATCTGC |
| rs1346004 | ACGTTGGATGCAGT  TCAGTTAAGGCAAGAC | ACGTTGGATGTTG  ATAAATCCCCCACCTTC | R | TCCCCCACCT  TCTGATAAC |
| rs4869739 | ACGTTGGATGGTG  GCTCTGAATAGCTTACC | ACGTTGGATGTTCC  ATGGCTCTCAAGCTTC | R | cCTGTGTGTG  TGCTTGAAA |
| rs1038304 | ACGTTGGATGCCTG  GCCAAAACTGGTTTCT | ACGTTGGATGCACTA  AATTGAGGAATCCAG | R | tgGAGGAATCC  AGAGTCAAATA |

PCR = polymerase chain reaction, UEP = un-extended mini-sequencing primer.
